# Imaging Features of Toxicities by Immune Checkpoint Inhibitors in Cancer Therapy

**DOI:** 10.1007/s40134-017-0256-2

**Published:** 2017-09-11

**Authors:** Gerlig Widmann, Van Anh Nguyen, Julian Plaickner, Werner Jaschke

**Affiliations:** 1 Department of Radiology, Medical University of Innsbruck, Anichstr. 35, 6020 Innsbruck, Austria; 2 Department of Dermatology, Venereology and Allergology, Medical University of Innsbruck, Anichstr. 35, 6020 Innsbruck, Austria

**Keywords:** Immune checkpoint inhibitors, Immune-related adverse events, Imaging

## Abstract

**Purpose of review:**

With the increasing use of immune checkpoint inhibitors in cancer therapy radiographic profiling of frequent and serious immune-related adverse events (irAEs) becomes more relevant. This article reviews imaging features of irAEs induced by the anti-CTLA-4 and anti-PD-1 antibodies ipilimumab, nivolumab and pembrolizumab.

**Recent findings:**

Important radiological manifestations are immune-related colitis, hepatitis, pancreatitis, hypophysitis, pneumonitis, arthritis and sarcoid-like lymphadenopathy. Typical imaging features are summarized and compared with other relevant differential diagnoses.

**Summary:**

Early diagnosis and appropriate therapeutic decisions are required for a successful treatment of irAEs. In addition to staging and follow-up imaging, identification and monitoring of adverse events becomes an important radiologic aspect in oncologic care.

## Introduction

The recent introduction of a targeted immunotherapy by a novel class of drugs named immune checkpoint inhibitors (ICPIs) has opened new treatment options for several cancer types, such as metastatic melanoma, non-small cell lung cancer, and renal cell cancer [1]. The basic principle of ICPI therapy is the inhibition of negative regulatory components of the T-cell-mediated immune response against cancer cells [2].

As a result of the impaired self-tolerance due to the loss of T-cell inhibition, ICPIs may cause a unique spectrum of immune-related adverse events (irAEs) [3••, 4]. IrAEs can affect any organ and system and may clinically manifest as colitis, hepatitis, pneumonitis, sarcoid-like lymphadenopathy and hypophysitis, which are detectable by computed tomography (CT) magnetic resonance tomography (MRT), or positron emission tomography/computed tomography (PET-CT) [5•]. Symptoms from ICPIs are frequent and may present as serious and life-threatening events, which require timely patient management and adequate therapeutic decisions [6]. Thus, in addition to staging and monitoring of oncologic treatment—using immune-related tumour response criteria (irRC)—radiologists will be in charge for diagnosis and monitoring of immune-related toxicities in a growing population of cancer patients receiving immunotherapeutic agents [7–9].

The aim of this article was to review the most relevant imaging features of irAEs induced by ipilimumab, nivolumab and pembrolizumab. Typical radiographic findings were summarized, supported by pictorial examples from our institution and compared with other possible differential diagnoses.

## Background

Ipilimumab was the first ICPI approved in 2011 for treatment of metastatic melanoma. It is a monoclonal antibody that blocks cytotoxic T-lymphocyte-associated antigen 4 (CTLA-4), a checkpoint receptor on T-cells that ligates to B7 molecules (CD80 and CD86) on antigen-presenting cells [2]. A second class of ICPIs are nivolumab and pembrolizumab, which are monoclonal antibodies that block the interaction between another checkpoint receptor on T-cells called cell death protein 1 (PD-1) and its ligands PD-L1 and PD-L2 on antigen-presenting cells and tumour cells [10]. Blocking of CTLA-4 and PD-1 mediated pathways results in activation and proliferation of effector T-cells and simultaneous depletion of regulatory T-cells [1, 2]. These mechanisms are important stimulators of the cell-mediated cytotoxic effect of the immune system against tumour cells and inhibitors of tumoural immune resistance [2, 11].

The incidence of side-effects of ICPIs is high and may reach up to 80–90% of patients [12]. Symptomatic irAEs may present with a broad clinical spectrum which can be graded according to Common Terminology Criteria for Adverse Events (CTCAE) as grade 1 (mild), grade 2 (moderate), grade 3 (severe) and grade 4 (life threatening) [13]. A recent review on published toxicity data from phase II and III studies summarized incidences of grade 3 and 4 irAEs of 20–30% for ipilimumab, 10–15% for nivolumab and pembrolizumab and 55% for the combination therapy of ipilimumab and nivolumab [3••]. Toxicities due to ipilimumab are dose dependent and show higher rates of gastrointestinal, dermatologic and endocrine irAEs [3••, 14]. In contrast, toxicities of nivolumab and pembrolizumab are dose independent and associated with higher rates of hepatologic and pulmonary irAEs [3••, 14]. The onset of irAEs after the initiation of ipilimumab may show a temporal association: (a) skin manifestations after 2–3 weeks (1st dose), (b) colitis after 5–10 weeks (2nd dose), (c) hepatitis after 12–16 weeks (3rd dose), (d) endocrine dysfunctions after 9 weeks (4th dose) and (e) pneumonitis after 8–14 weeks [14]. Some patients may simultaneously have more than one irAE [15]. Management of irAEs is based on clinical judgement and follows the CTCAE guidelines [3••, 14]. As a general strategy, ICPIs are interrupted in grade 2 toxicities and resumed when symptoms decrease below grade 1. For grade 3 and 4 high dose glucocorticoids are given, with additional immunosuppressive agents if symptoms cannot be controlled, and ICPIs are permanently discontinued in patients with grade 4 toxicities. A more detailed description of treatment options would go beyond the scope of this review.

 Radiologic manifestations of irAEs may include colitis, hepatitis, pancreatitis, hypophysitis, thyroiditis, pneumonitis, arthritis, sarcoid-like lymphadenopathy and inflammatory changes in the soft tissues (myositis, fasciitis and retroperitoneal fat haziness) [5•, 8, 16•]. Published incidences of radiologic abnormalities obtained from retrospective institutional reviews of patients treated with ipilimumab for metastatic melanoma may amount to 31% [5•, 16•]. An association between incidence of irAEs and clinical response is suggested [5•]. In scans following treatment of irAEs up to 90% of imaging findings have resolved [5•, 16•].

## Colitis

Besides dermatologic manifestation such as pruritus and rash, gastrointestinal toxicities have been identified as the most frequent irAEs [4]. Diarrhoea and colitis typically occur 5 weeks after onset of therapy and show a prevalence of 36–38% and 8–10% for ipilimumab and 8–20% and 1–3% for nivolumab and prembolizumab, respectively [14]. Grade 3 and 4 colitis are seen in 7–9% of patients receiving ipilimumab and in approximately 1–2% for the group with nivolumab and prembolizumab [3••, 14]. Serious and life-threatening events may result from significant bloody diarrhoea and intestinal perforation [4, 17, 18].

**Fig. 1:**
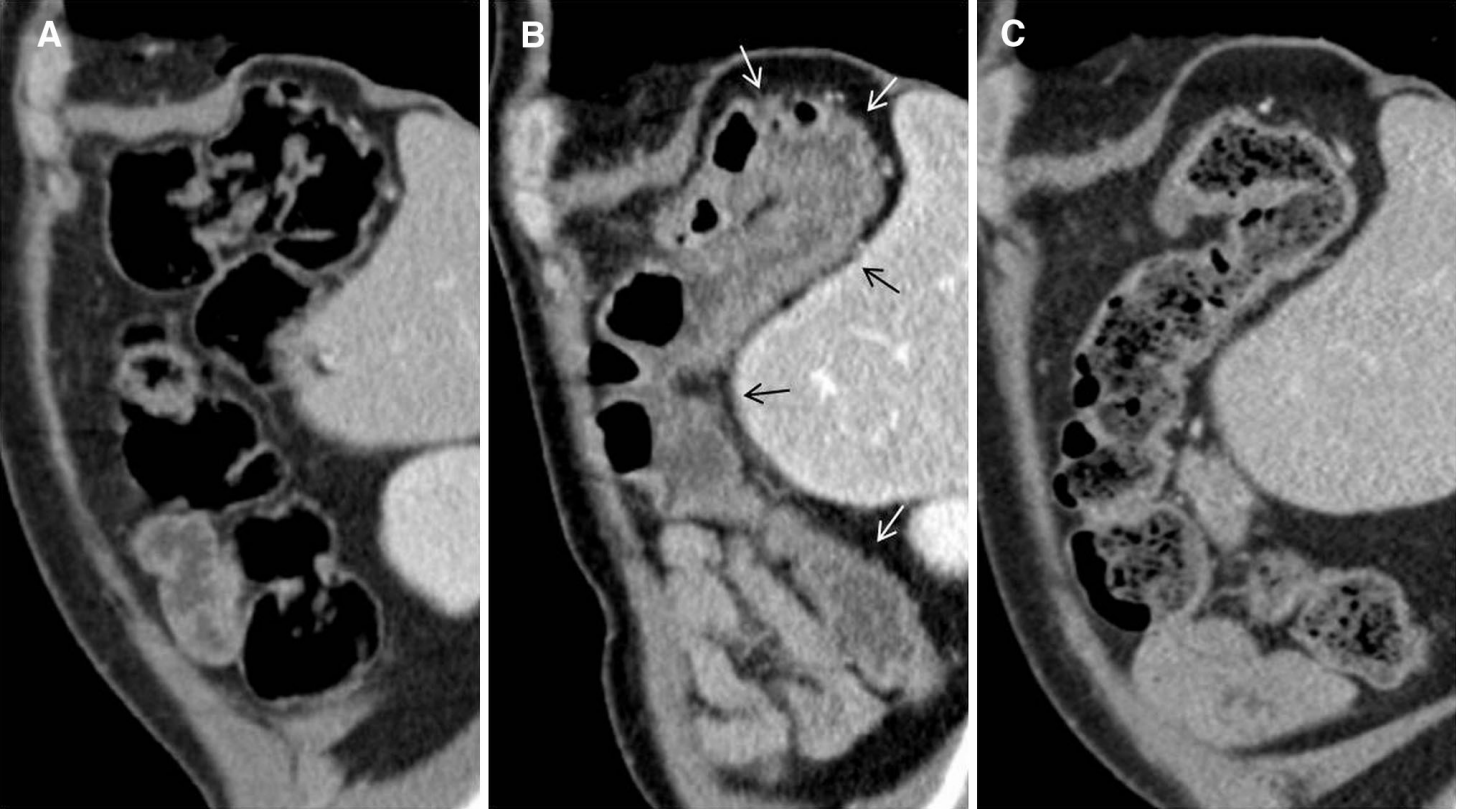
72-year-old man with colitis grade 2 after 4th dose of ipilimumab. **a** Normal colon before ipilimumab. **b** Colitis with diffuse colitis pattern. Colonic wall thickening and mucosal hyperenhancement (*arrows*). **c** Improvement after ipilimumab interruption and glucocorticoids

In a retrospective case-series reporting 16 patients with ipilimumab associated colitis with available CT evaluation, Kim et al. [17] found mesenteric vessel engorgement in 83%, bowel wall thickening in 75%, and fluid-filled colonic distension in 25%. Pericolic fat stranding was seen in 16% of patients in the case-series reported by Barina et al. [19]. In both publications there were no patients with pneumatosis or pneumoperitoneum [17, 19]. Immune-related colitis may present in three different patterns (see Table 1 and example in Fig. 1): (a) diffuse colitis, (b) segmental colitis associated with diverticulitis and (c) isolated recto-sigmoid colitis without diverticulosis [19]. Colonic wall thickness was greater and pericolic fat stranding was more frequently observed in the segmental colitis associated with diverticulitis pattern than in the diffuse colitis pattern [17]. Clinical symptoms with mixed watery and bloody diarrhoea and cramping pain were more severe in the segmental colitis associated with diverticulitis pattern, whereas diffuse colitis and isolated recto-sigmoid colitis without diverticulosis pattern seemed less symptomatic with a predominance of watery diarrhoea [17, 19]. Presence of an ipilimumab induced ileitis without colits is extremely rare and has been described in one case report [20].

**Table 1:** Typical imaging findings of immune-related adverse events (irAEs) and important differential diagnoses

irAE	Typical imaging findings	Important differential diagnoses
Colitis	Diffuse colitis pattern: diffuse colonic wall thickening, mucosal hyperenhancement, mesenteric vessel engorgement Segmental colitis associated with diverticulosis pattern: Colonic wall thickening, pericolic fat stranding, mesenteric vessel engorgement, mucosal hyperenhancement, diverticula Isolated recto-sigmoid colitis without diverticulosis pattern: Colonic wall thickening, mucosal hyperenhancement, pericolic fat stranding	Crohn’s disease: predominately involves the terminal ileum, patchy transmural distribution, inflammatory stranding, submucosal fat, fibrofatty hyperplasia Ulcerative colitis: Affects the colon with increasing intensity distally, Diffuse crypt atrophy, submucosal halo of fat Infectious colitis: Most commonly limited to the right colon, wall thickening, homogeneous enhancement Pseudomembranous colitis: Marked circumferential or eccentric colonic wall thickening
Hepatitis	CT, MRT: hepatomegaly, periportal oedema, attenuated liver parenchyma, periportal lymphadenopathy US: prominent periportal echogenicity, gallbladder wall oedema	Viral hepatitis: Positive viral serology Acute alcoholic liver disease: History of alcohol abuse, steatohepatitis, cirrhosis Idiopathic autoimmune hepatitis: Cirrhosis, autoantibodies
Pancreatitis	CT, MR: pancreatic enlargement, decreased enhancement, surrounding fat stranding PET-CT: increased FDG uptake	Immunoglobulin G4-related disease: Loss of the normal fatty lobulations ‘sausage pancreas’, may show focal forms, may include simultaneous findings in multiple other organs (e.g. biliary, salivary, aortic, retroperitoneal), MRCP: diffused narrow or segmental stenosis of the main pancreatic duct (‘pancreatic duct penetrating sign’) without an upstream dilation, may show strictures of the pancreatic segment of common bile duct, proximal bile duct dilation, gallbladder enlargement
Pneumonitis	Patterns: Cryptogenic organizing pneumonia (COP) > non-specific interstitial pneumonia (NSIP) > hypersensitivity pneumonitis (HP) and acute interstitial pneumonia/acute respiratory distress syndrome (AIP/ARDS) Involvement: Lower > middle > upper lungs Distribution: Mixed and multifocal > peripheral and lower and diffuse Specific findings: Ground glass opacities, reticular opacities, consolidations	Bacterial pneumonia: Consolidations with air-bronchogram, pleural effusion Drug-induced pneumonitis: Review of medication history Radiation pneumonitis: Involves the lung area that is exposed by a radiation dose above a threshold of 30-40 Gray, Not limited by anatomical borders such as interlobar fissures and bronchovascular structures, shows ground glass opacities which may increase in density and consolidate over time
Hypophysitis	Moderate symmetric enlargement of the pituitary, convex aspect, enlargement of the stalk or infundibulum, homogeneous contrast enhancement	Pituitary adenoma: Asymmetric enlargement, heterogeneous contrast enhancement, loss of pituitary bright spot Pituitary metastasis: Melanoma, breast, and lung cancer (rare) Lymphocytic hypophysitis: Young women during pregnancy or postpartum period with headache, visual impairment, and ACTH deficiency
Arthritis	US and MRT: proliferative synovitis (hyperaemia and synovial thickening), joint effusions, sometimes joint erosions, tenosynovitis, bone marrow edema, myositis PET-CT: Increased FDG uptake in the synovia of multiple bilateral joints Pattern: Non-specific, large and small joints, may present as oligoarthritis, additive arthritis, or severe polyarthritis	Rheumatoid arthritis: Symmetrical small joint involvement, metacarpophalangeal and proximal interphalangeal joints, rheumatoid factor, anti-cyclic citrullinated peptide antibodies
Sarcoid-like lymphadenopathy	Symmetric mediastinal and hilar lymph enlargement with either new lymph nodes or enlargement of pre-existing nodes	Metastatic lymphadenopathy: May be asymmetric, may show inhomogeneous enhancement with natural or treatment related necrosis

Important differential diagnoses of immune mediated colitis are Crohn’s Disease, ulcerative colitis, infectious and pseudomembranous colitis (see Table 1). Crohn’s disease predominately involves the terminal ileum and has a patchy transmural distribution, ulcerative colitis typically affects the colon with increasing intensity distally, infectious colitis is most commonly limited to the right colon and pseudomembranous colitis shows a marked circumferential or eccentric wall thickening throughout the colon [21, 22].

## Hepatitis

Immune-related hepatotoxic effects leading to elevation of hepatic transaminases with or without bilirubin may be observed in 1–10% of patients on monotherapy but up to 30% of patients receiving combination therapy of nivolumab plus ipilimumab [3••, 14]. Grade 3 toxicity with elevated hepatic transaminases 3–5 times the upper limit of normal has been recorded in around 1–2% of patients receiving monotherapy and 14% of patients treated with the combination [3••]. Patients are usually asymptomatic but may sometimes present with fever, fatigue, jaundice and changes of stool colour [22].

**Fig. 2:**
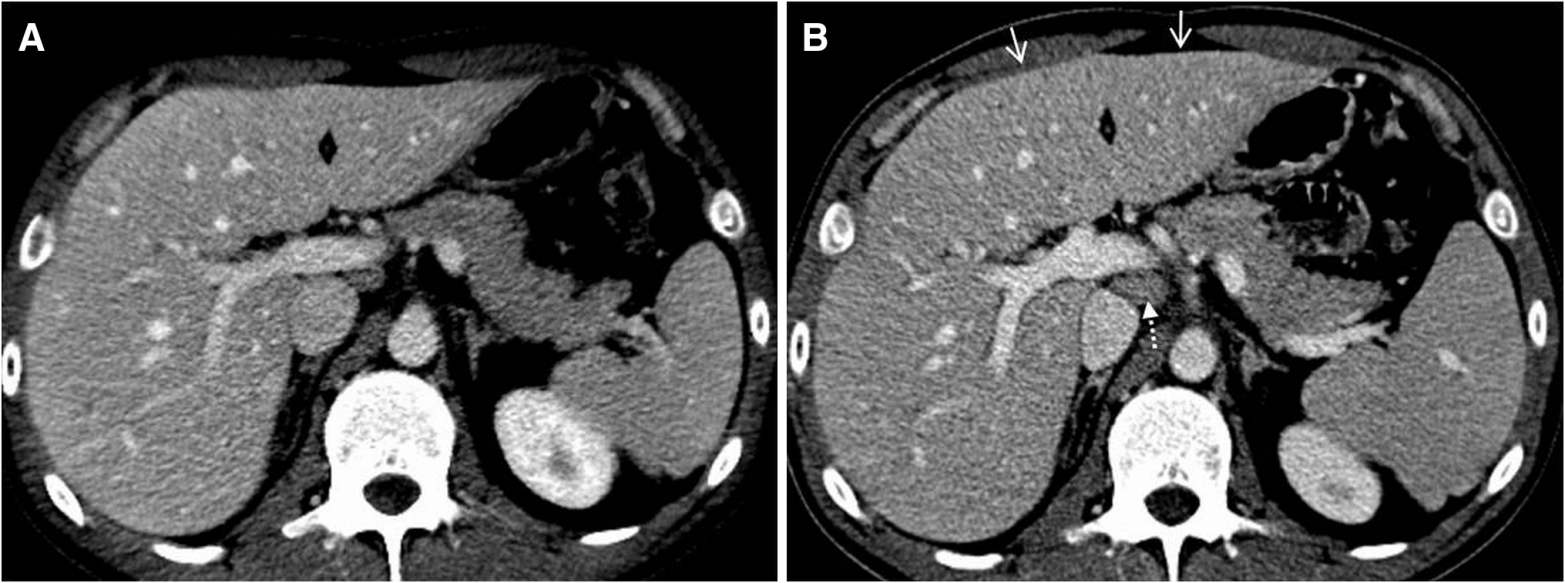
33-year-old man with hepatitis grade 3 after 3rd dose of nivolumab plus ipilimumab. **a** Normal sized liver before combination therapy. **b** Hepatis with convex shaped hepatomegaly (*arrows*) and periportal lymphadenopathy (*dotted arrow*)

On CT and MRT a manifest immune-related hepatitis may present with hepatomegaly, periportal oedema, and attenuated liver parenchyma compared with the baseline CT, periportal T2-hyperintensity on MRT, and enlarged periportal lymph nodes (see Table 1; Fig. 2) [7, 16•, 23]. On ultrasound (US), prominent periportal echogenicity and gallbladder wall oedema were described [22, 23]. In a case-series of 6 patients with ipilimumab associated hepatitis, Kim et al. [23] reported that a decreased attenuation may obscure hepatic metastases and that new geographic areas of low-attenuation may mimic metastases. On follow-up imaging after glucocorticoid therapy hepatomegaly and periportal lymphadenopathy usually resolve [23].

Imaging findings are non-specific and may be similar to that of viral and alcohol induced acute hepatitis, and idiopathic autoimmune hepatitis [24]. Further, extrahepatic cholestasis and new onset of metastatic liver disease have to be ruled out [23].

## Pancreatitis

ICPI related pancreatitis is rare with an incidence of <1% [16•]. It is associated with increase in serum amylase and lipase, may be clinically asymptomatic or present with upper abdominal pain [14].

**Fig. 3:**
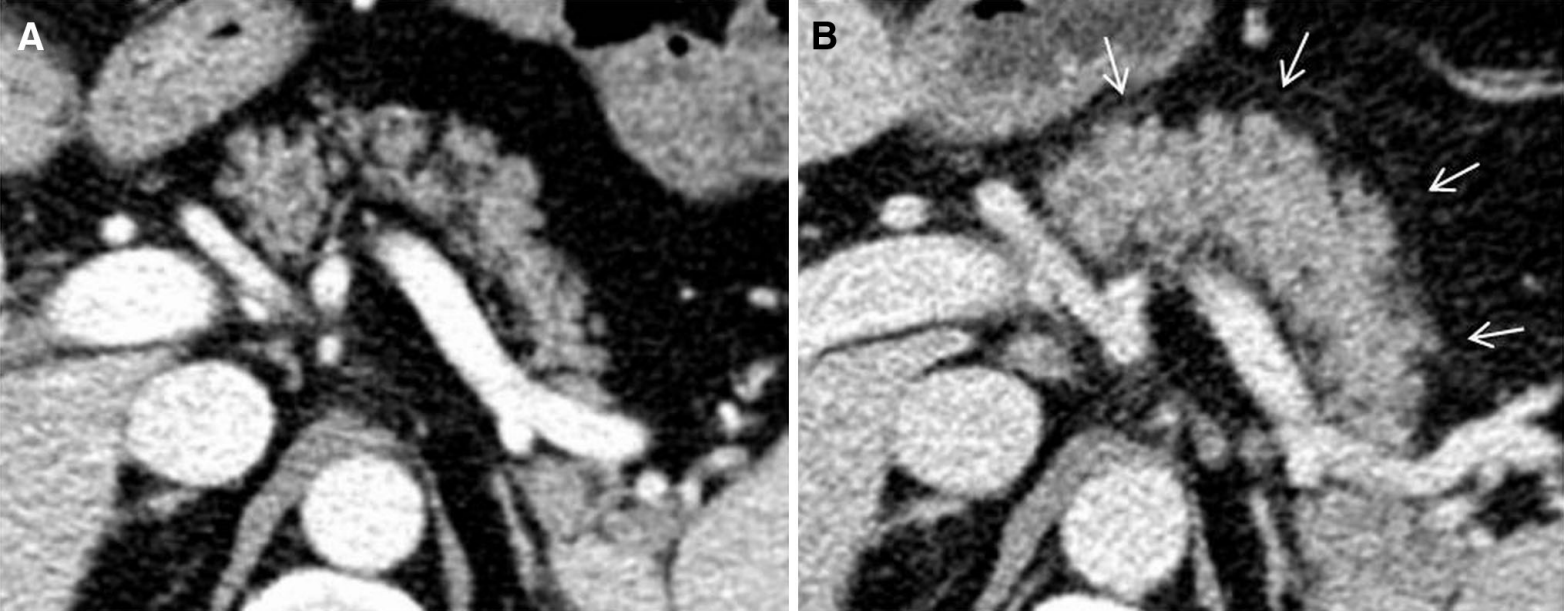
54-year-old man with pancreatitis after 1st dose of pembrolizumab. **a** Normal pancreas before pembrolizumab. **b** Pancreatitis with pancreatic enlargement and fat stranding (*arrows*)

On CT and MRT, pancreatic enlargement, decrease in attenuation and surrounding fat stranding can be seen (see Table 1; Fig. 3) [16•]. PET-CT may show intense fluor deoxy glucose (FDG) FDG uptake in the pancreas [9]. The findings may be difficult to distinguish from immunoglobulin G4-related autoimmune pancreatitis [9, 25]. However, the latter may include focal forms, may show a typical loss of the normal fatty lobulations described as a ‘sausage pancreas’ and may include simultaneous findings in multiple other organs (such as biliary, salivary, aortic and retroperitoneal involvement) [26, 27]. Magnetic resonance cholangiopancreatography (MRCP) may show diffused narrow or segmental stenosis of the main pancreatic duct (‘pancreatic duct penetrating sign’) without an upstream dilation, strictures of the pancreatic segment of common bile duct, proximal bile duct dilation and gallbladder enlargement [25, 27, 28]. In addition, immunoglobulin G4-related autoimmune pancreatitis often presents with obstructive jaundice and upper abdominal pain [26].

## Pneumonitis

Pulmonary toxicities are rare and typically occur late after about 10 months from the start of ICPI treatment [14]. Symptoms may range from asymptomatic and mild in grade 1 and 2 to cough, hypoxia and life-threatening respiratory failure in grade 3 and 4, requiring hospitalization and immediate intervention, respectively [6, 14]. Pneumonitis has been more frequently reported in patients treated for advanced lung cancer (3–5%) than in patients for malign melanoma (0–2%) [3••]. Prevalence is higher for nivolumab and pembrolizumab and the nivolumab/ipilimumab combined therapy than for ipilimumab monotherapy [12, 29]. Patients with pre-existing lung disease and who received radiotherapy of lung metastases before ICPI therapy may have an increased risk for developing immune-related pneumonitis [30, 31].

**Fig. 4:**
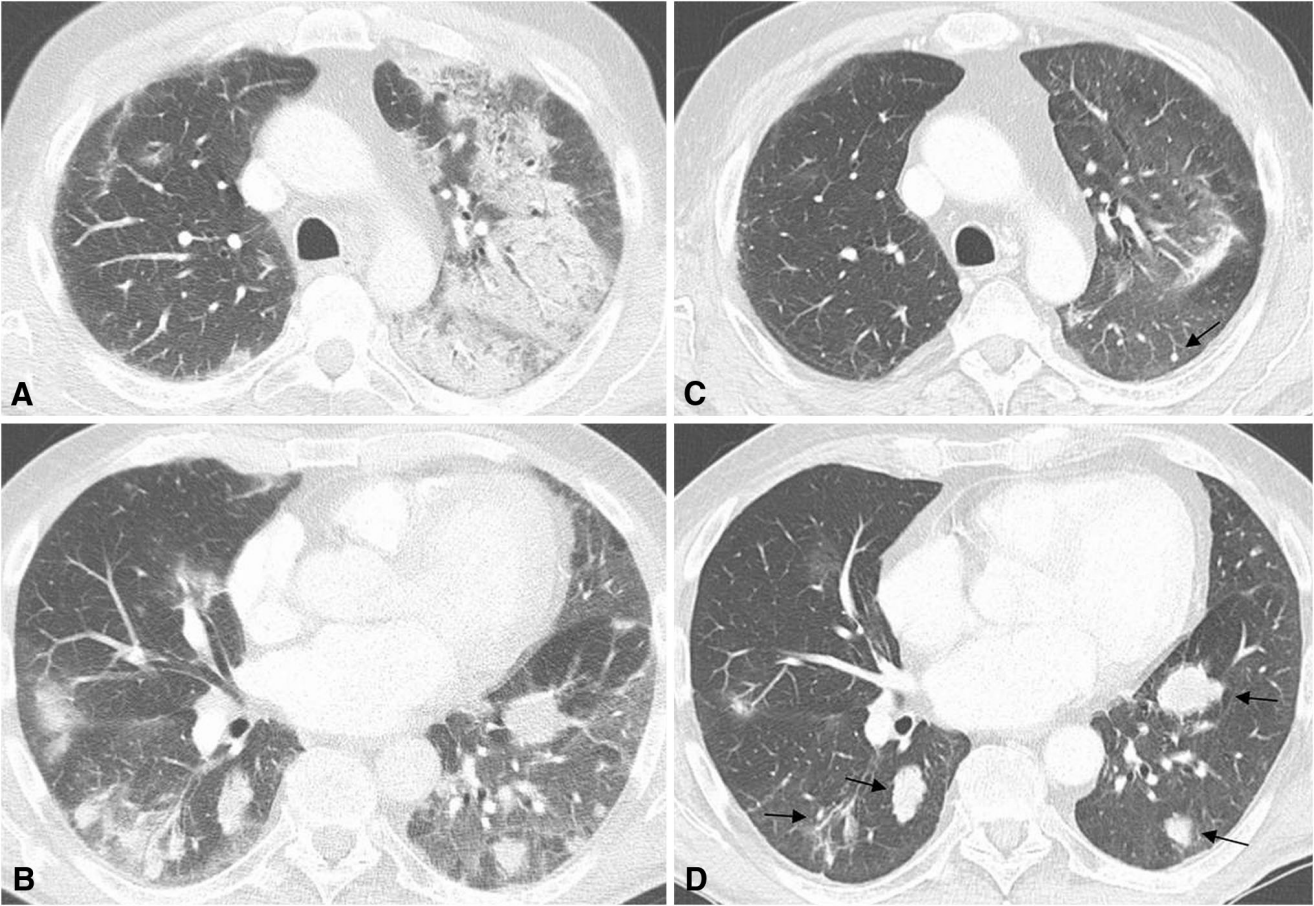
73-year-old man with pneumonitis grade 3 after 3rd dose of ipilimumab given sequentially after 9 doses of pembrolizumab due to disease progression. **a** and **b** Pneumonitis with cryptogenic organizing pneumonia pattern. Mixed and multifocal peripheral ground glass opacities and consolidations. **c** and **d** Improvement after ipilimumab interruption and glucocorticoids. *Arrows* show multiple lung metastases

Following the classification according to the American Thoracic Society/European Respiratory Society classification of interstitial pneumonias, Nishino et al. [32] summarized the following imaging findings obtained from 20 patients with PD1-related pneumonitis: a) cryptogenic organizing pneumonia (COP) pattern in 65%, b) non-specific interstitial pneumonia pattern (NSIP) in 15%, c) hypersensitivity pneumonitis (HP) pattern in 10% and d) acute interstitial pneumonia/acute respiratory distress syndrome (AIP/ARDS) pattern in 10%. The lower lungs were more frequently involved than the middle and upper lungs and mixed and multifocal distributions were dominant over peripheral and lower and diffuse distributions [32]. Ground glass opacities were found in all, and reticular opacities and consolidations in most of the cases [32]. Table 1 shows a summary of these findings and an example is given in Fig. 4. A’flare’ of clinical and imaging signs of recurrent pneumonitis may occur after taper of glucocorticoid therapy [32, 33].

Imaging may help ruling out other pulmonary diseases such as bacterial pneumonia, which typically show asymmetrical consolidations with air-bronchogram and pleural effusion [34]. In addition, resistance to antibiotic treatment, negative sputum, bronchioalveolar lavage and pleural fluid culture may be important clues to support the diagnosis of immune-related pneumonitis [34]. To distinguish pulmonary toxicities from cytotoxic and non-cytotoxic drugs a careful review of the patient’s medication history is required [35]. Radiation pneumonitis usually involves the lung area that is exposed by a radiation dose above a threshold of 30–40 Gray and is not limited by anatomical borders such as interlobar fissures and bronchovascular structures [36]. Onset is after about 6–10 weeks and typical CT findings are ground glass opacities which may increase in density and consolidate over time [36].

## Hypophysitis

Hypophysitis has been reported as a complication of ipilimumab therapy [14]. It is synonymously named as ipilimumab induced hypophysitis (IIH) or ipilimumab associated autoimmune hypophysitis (IAH) [37]. Hypophysitis usually emerges after the third cycle of ipilimumab about 9 weeks from start of treatment and has an incidence of 2–4% [14]. Higher incidences of 8% were reported by Larkin et al. [38] for combined nivolumab plus ipilimumab and up to 25% by Albarel et al. [39] for patients receiving higher dose regimen of ipilimumab (10 mg/kg). Initial symptoms are headache and fatigue, followed by hypothyroidism, hypogonadism and hypocortisolism, which can be serious and life threatening [14, 37, 39].

**Fig. 5:**
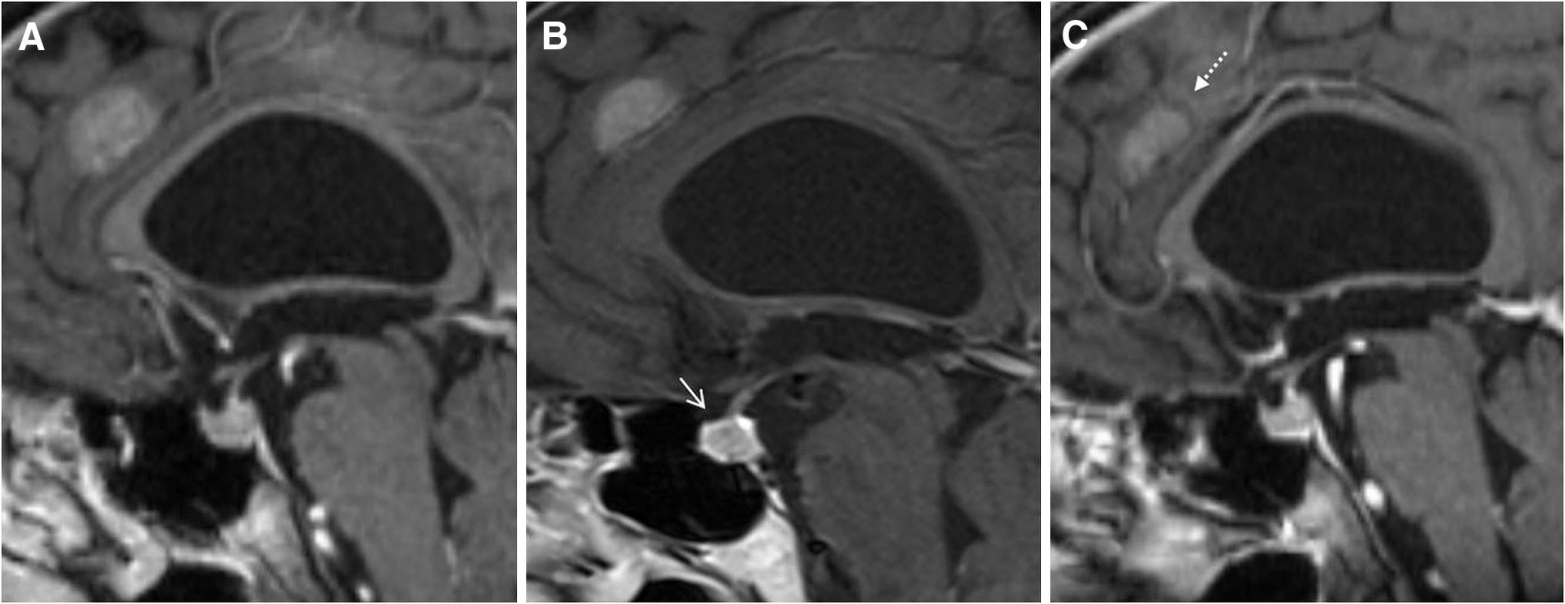
73-year-old man with hypophysitits grade 3 after 5th dose of ipilimumab. **a** Normal pituitary gland before ipilimumab. **b** Hypophysitis with moderate enlargement of the pituitary and homogeneous contrast enhancement (*arrow*). **c** Recovery of pituitary gland to normal size after ipilimumab interruption and glucocorticoids. *Dotted arrow* shows brain metastasis with partial regression

In the imaging review by Araujo et al. [37] which included 57 cases of ipilimumab associated hypophysitis, pathologic findings of the pituitary gland were observed in 77%. Typical MRT findings are moderate enlargement of the pituitary, convex aspect, enlargement of the stalk or infundibulum and homogeneous contrast enhancement (see Table 1; Fig. 5) [37, 39]. Heterogeneous contrast enhancement has been reported as an untypical feature [40]. Marlier et al. [41] found an enlargement of the pituitary gland only in the two cases receiving 10 mg/kg of ipilimumab, whereas the other symptomatic two cases receiving 3 mg/kg of ipilimumab showed a normal size.

Follow-up MRT after glucocorticoid treatment may demonstrate a decrease in pituitary volume, a change of aspect from convex to concave or in extreme an empty sella [39]. The majority of patients (64–76%) may show no pituitary function recovery [37, 42]. Immune-related thyroiditis which usually leads to hypothyroidism may occur either isolated or concurrent with hypophysitis and can be detected by increased fluor deoxy glucose (FDG) uptake on PET-CT) [16•].

Important differential diagnoses of ipilimumab associated hypophysitis are pituitary macroadenomas, new onset of brain metastasis and rare pituitary metastasis from melanoma, breast and lung cancer [37]. In contrast to hypophysitis, macroadenomas are usually associated with an asymmetric or focally enlarged pituitary with normal stalk and show a heterogenous enhancement without dural tail [40]. Autoimmune pituitary disease such as lymphocytic hypophysitis may show identical findings to ipilimumab associated hypophysitis [43, 44]. However, it predominantly affects young women during pregnancy or postpartum period with headache, visual impairment and ACTH deficiency [43].

## Arthritis

Rheumatological side-effects from ICPIs may include arthralgia, myalgia, inflammatory arthritis and myositis [12]. The incidence of arthralgia is around 9–12% and 6–8% for patients receiving pembrolizumab and nivolumab, 5% for patients receiving ipilimumab and 11% for patients receiving the combination therapy of nivolumab and ipilimumab [3••]. Manifest arthritis is less frequently reported and may affect 2% of patients on anti-PD-1 inhibitors [3••, 31]. In a case-series of Cappelli et al. [15] including 13 patients with ICPI related rheumatological events, inflammatory arthritis was noted in 9 patients with imaging confirmed synovitis and inflammatory synovial fluid in 4 patients. Inflammatory arthritis may affect both large and small joints, and may present as oligoarthritis, additive arthritis or severe polyarthritis [3••, 15, 31].

**Fig. 6:**
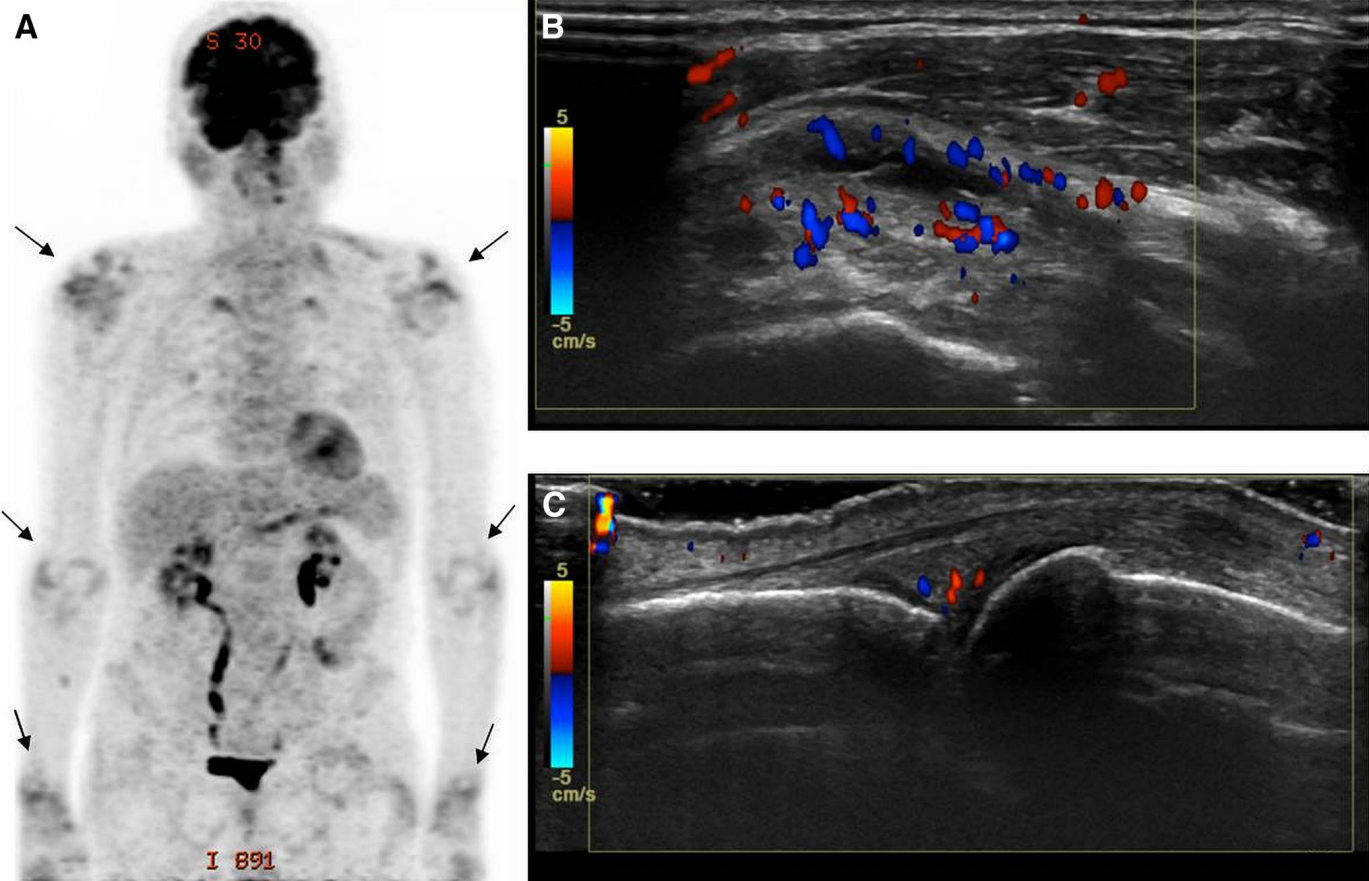
76-year-old man with arthritis after 21st dose of nivolumab. **a** PET-CT shows oligoarthritis with increased FDG uptake in the shoulders, elbows and wrists (*arrows*). **b** Shoulder arthritis with thickened synovial and increased vascularity. **c** Metacarpophalangeal arthritis with thickened increased vascularized synovia

Imaging findings on US and MRT are proliferative synovitis with typical hyperaemia and synovial thickening, joint effusions and sometimes joint erosions, tenosynovitis, bone marrow edema and myositis (see Table 1; Fig. 6) [15, 45]. On PET-CT, increased FDG uptake in the synovia of multiple bilateral joints and in muscles may be observed (Fig. 6) [5•, 8, 9]. Differentiation from rheumatoid arthritis can be very difficult; however it has been reported that anti-cyclic citrullinated peptide antibodies and rheumatoid factors are usually absent [15]. Suarez-Almazor et al. [31] postulated two potential pathophysiological explanations for ICPI related arthritis, with one group of patients developing a non-specific arthritis due to up-regulation of the immune system and another group with onset of rheumatoid arthritis bases on a genetic or environmental predisposition.

## Sarcoid-Like Lymphadenopathy

**Fig. 7:**
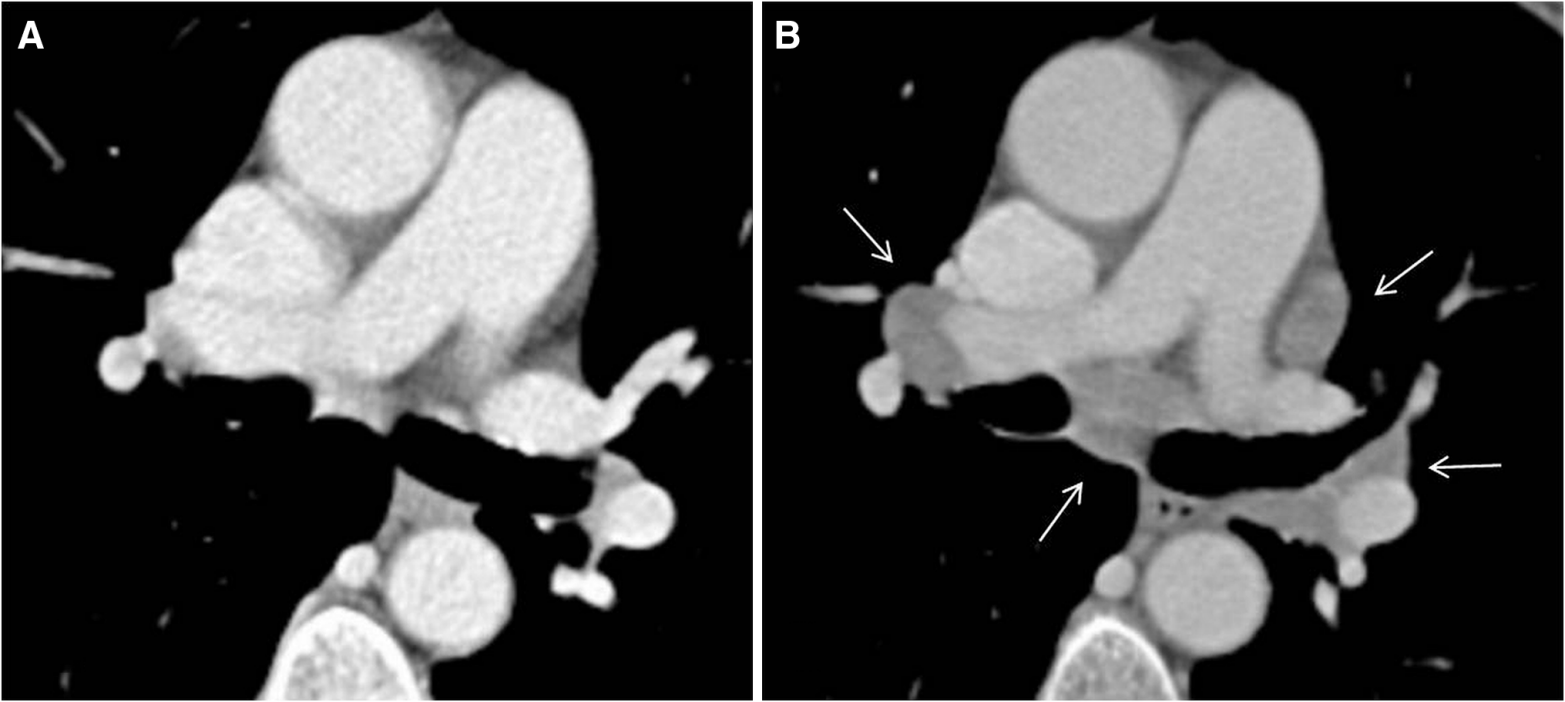
67-year-old woman with sarcoid lymphadenopathy after 9th dose of pembrolizumab. **a** Normal mediastinal and hilar lymph nodes before pembrolizumab. **b** Sarcoid lymphadenopathy (biopsy confirmed) with symmetric hilar and mediastinal enlargement of lymph nodes

Immune-related sarcoid-like lymphadenopathy has been described as an asymptomatic radiographic finding in around 5–7% of patients [5•, 8, 16•]. It may present as new onset of symmetric mediastinal and hilar lymph enlargement, with either new lymph nodes or enlargement of pre-existing nodes (see Table 1; Fig. 7) [5•]. Tirumani et al. [16•] reported various coexisting pulmonary findings such as bilateral irregular nodular and patchy opacities, or ground glass and interstitial opacities in 3 of 8 patients. Lymphadenopathy may resolve in most patients [5•, 16•].

Differentiation of sarcoid-like lymphadenopathy from metastatic and reactive lymph nodes may be extremely difficult as imaging findings can be unspecific and misleading [8]. Metastatic lymph nodes may show inhomogenous contrast enhancement and necrosis related to natural or treatment related necrosis [5•].

## Conclusions

Without doubt, with the increasing use of ICPIs in a growing number of tumour types, incidences of immune-related toxicities will increase. The most important radiologic manifestations of irAE induced by anti-CTLA-4 and anti-PD-1 antibodies may include colitis, hepatitis, pancreatitis, hypophysitis, pneumonitis, arthritis and sarcoid-like lymphadenopathy. Imaging findings of toxic effects from newer ICPIs such as atezolizumab, tremelimumab and pidilizumab may be similar but were not included in this review. Knowledge of typical radiographic features of irAEs is essential to manage early diagnosis and to rule out other relevant differential diagnoses including disease progression. Series and life-threatening events may result from colitis, pneumonitis and hypophysitis. The differentiation of irAEs from autoimmune disorders and immune-related pneumonitis from infectious and drug-induced findings can be very difficult and requires a close multidisciplinary clinical collaboration. With prompt initiation of therapy according to clinical grades most irAEs can be successfully treated. Identification and monitoring of treatment related toxicities increase radiologic responsibility in oncologic care.
